# Radiological Presentations of Prostatic Carcinoma Patients: A Retrospective Observational Study From a Tertiary Care Oncology Setup

**DOI:** 10.7759/cureus.107876

**Published:** 2026-04-28

**Authors:** Muhammad O Altaf, Khadija Nasir, Zunairah Mughal, Muhammad M Niaz

**Affiliations:** 1 Department of Radiology, Shaukat Khanum Memorial Cancer Hospital and Research Centre, Lahore, PAK; 2 Department of Radiology, Evercare Hospital, Lahore, PAK; 3 Department of Radiology, Shalamar Hospital, Lahore, PAK

**Keywords:** gleason scoring, lymph node, metastasis, prostate cancer, prostate-specific antigen (psa), radiology

## Abstract

Introduction

Prostate cancer is the second most common cancer in men. Accurate staging is necessary for optimal planning before treatment. Radiological imaging has a significant role in the evaluation of tumor detection, lymph node involvement, and distant metastasis. Despite the advancements in diagnostic radiological imaging, there is still insufficient local data addressing the radiological spectrum of prostate cancer presentations and their link to clinical and pathological factors.

Methodology

A retrospective cross-sectional analysis was performed at the Shaukat Khanum Memorial Cancer Hospital and Research Centre, Lahore, Pakistan. The study included histopathologically confirmed prostate cancer cases presenting between January 2015 and December 2019. Computed tomography (CT) and magnetic resonance imaging (MRI) findings were reviewed. TNM staging, prostate-specific antigen (PSA) levels, and Gleason scores were recorded. Data analysis was conducted using SPSS version 26 (IBM Inc., Armonk, New York), utilizing univariate and multivariate regression to determine the associations between demographic and clinical variables with metastasis and lymph node involvement.

Results

Only 203 patients were included, with a mean age of 65.13 ± 6.84 years. The majority of the participants were >65 years old (117; 57.6%), PSA level of 4-10 ng/ml (258; 77.8%), and Gleason score of 8-10 (121; 59.6%). The most common radiological stage was T3b (104; 51.2%). Metastasis and lymph node involvement were present in 120 (59.1%) and 87 (42.9%), respectively. Bones (97; 47.8%) were the predominant site of primary metastasis. PSA level 10ng/ml and high Gleason score had a significant association with metastasis.

Conclusion

Most patients presented with advanced-stage prostate cancer, lymph node involvement, and metastases. Elevated PSA levels and high Gleason scores were significantly associated with the presence of metastasis. These findings highlight the necessity for earlier detection through enhanced awareness and screening of prostate cancer.

## Introduction

Prostate cancer is the fourth most common cancer and the second most common cancer in men worldwide. The new cases of prostate cancer reported were 1,467,854 in 2022, with the highest incidence in the USA. It is the fifth leading cause of cancer-related mortality and accounts for 397,430 deaths in 2022 [1]. Age is an independent risk factor for prostate cancer, and it is rare in the younger age group. The incidence of prostate cancer rises significantly after 55 years age of and 66 years is the usual age of diagnosis [2,3]. Incidence and mortality rates of prostate cancer vary considerably across the geographical regions. The difference in the epidemiological distribution of prostate cancer depends on the genetic predisposition, ethnicity, and family history, with the inclusion of individual, environmental, and occupational risk factors. The diversity in health and socioeconomic status, coupled with varying healthcare policies in different countries, may contribute to the observed discrepancies in incidence and mortality [4]. Prostate cancer has an incidence rate of 11.2% in Southeast Asia, 10.5% in Eastern Asia, and 4.5% in South Central Asia [3]. Prostate cancer is also the second most common cancer in men in Pakistan, followed by colorectal cancer, with a prevalence rate of 8.5% [5].

Prostate cancer management requires the accurate detection and evaluation of the extent of the tumor. Staging of prostate cancer begins with the identification of the primary prostate lesion. It involves the assessment of the extent of the tumor, lymph node, and distant metastasis, providing the critical clinical information for both prognosis and predictability of disease, as well as treatment of prostate cancer. Various radiological imaging techniques, such as ultrasound (US), computed tomography (CT), and magnetic resonance imaging (MRI), have been employed for staging prostate cancer, each exhibiting differing levels of diagnostic efficacy [6]. The US and CT have been considered important investigations for staging prostate cancer with inherent limitations. The US is observer-dependent, and CT has limited capability for soft tissue enhancement [7,8]. MRI provides excellent soft-tissue differentiation and is considered the most accurate imaging modality for T-staging of prostate cancer [9]. A multiparametric imaging approach is recommended and comprises anatomical sequences such as T1-weighted (T1W) and T2-weighted (T2W) images, as well as functional sequences, including diffusion-weighted imaging (DWI) and dynamic contrast-enhanced (DCE) MRI [6].

Prostate-specific antigen (PSA) is an enzyme produced by the prostate gland and could rise in prostatic inflammation, benign prostatic hyperplasia, old age, and prostate cancer. Low levels of PSA do not eliminate the possibility of prostate cancer, and an increase in PSA levels alone is inadequate for a definitive diagnosis. PSA screening has contributed to a reduction in prostate cancer-related mortality; however, it is estimated that 20% and 60% of prostate cancer diagnoses identified through PSA screening are cases of overdiagnosis [10]. The integration of radiological imaging, PSA screening, and histopathological classification through Gleason scoring could reduce the burden of overdiagnosis and could contribute to accurate diagnosis, risk stratification, and management of prostate cancer.

The incidence of prostate cancer in Pakistan has been on the rise, with recent meta-analyses and registry data indicating a growing pooled prevalence and regional disparities among provinces. A systematic review and meta-analysis conducted in 2023 highlighted an increasing pooled prevalence of prostate cancer in Pakistan (5.20%), with notably higher estimates observed in provinces such as Khyber Pakhtunkhwa (8.29%) and Punjab (8.09%) [3]. A high proportion of patients present with locally advanced and metastatic disease at the time of presentation, indicating delayed detection and low screening uptake [11]. Poor awareness of prostate cancer and low participation in PSA/DRE screening programs in Pakistan contribute to late presentation [12].

Despite the recognized epidemiologic burden, there is limited published local data specifically describing the radiological spectrum of prostate cancer at presentation and its relationship to PSA levels and Gleason score in Pakistani cohorts. Radiology is central to accurate staging and treatment planning, but the extent to which imaging correlates with PSA and Gleason groups in routine practice in Pakistani tertiary centres has not been well characterized. By correlating radiological findings to PSA levels, Gleason scores, and metastasis, this study aims to provide a comprehensive understanding of the prostate carcinoma presentation at the time of diagnosis at Shaukat Khanum Memorial and Research Care Centre (SKMC), a tertiary care oncology hospital in Pakistan.

## Materials and methods

Study design and setting

A retrospective observational cohort study was conducted at the Department of Radiology, Shaukat Khanum Cancer and Memorial Hospital, Lahore, Pakistan. The patients diagnosed with prostate cancer on histopathology, who presented from January 2015 to December 2019, were included in the study. Patients who had received chemotherapy or radiotherapy before radiological imaging and an incomplete staging workup were excluded.

Ethical considerations

The retrospective study design excluded the requirement of informed consent from the participants, and no personal information as name, address, or contact information, was recorded for the study. The ethical approval of this study was acquired from the institutional review board of Shaukat Khanum Cancer and Memorial Hospital, Lahore, Pakistan.

Data collection

The data of all patients presenting with prostate cancer diagnosed on histopathological analysis were reviewed thoroughly, including presenting complaints, investigations, and previous medical and treatment history. Age, prostate-specific antigen (PSA) level, and Gleason score of all the patients were recorded. The contrast-enhanced computed tomography (CT) of the chest, abdomen, and pelvis performed on 64- and 160-slice Toshiba scanners of each patient was analyzed. Furthermore, MRI pelvis acquired on 1.5 T and 3 T scanners were also analyzed. Staging of the disease at presentation, following the American Joint Committee on Cancer (AJCC) guidelines of TNM staging, was done. Details, including the size of the tumor, margins, invasion, metastasis, vessel, and lymph node involvement, were noted. The two different radiologists reviewed the radiological investigations to minimize the observational bias. All the data was recorded on a pre-designed proforma.

Data analysis

Data was initially shifted to Microsoft Excel and was analyzed using Statistical Package for Social Sciences version 26 (IBM Inc., Armonk, New York). Patients were categorized by age as ≤ 65 and > 65 years, PSA level as <4, 4-10, and >10 ng/ml, and Gleason score as low grade (6), intermediate grade (7), and high grade (8-10). Quantitative variables were presented as mean ± standard deviation. Qualitative variables were presented as frequency and percentage. Univariate and multivariate regression analysis were utilized for the correlation of age, PSA level, and Gleason score with metastasis and lymph node involvement. The value of p<0.05 was considered statistically significant.

## Results

The study included 203 participants with a mean age, PSA level, and Gleason score of 65.13 ± 6.84 years, 213.39 ± 543.20 ng/ml, and 8.01 ± 1.65, respectively. The majority of the participants had an age >65 years (57.6%), a PSA level of more than 10 ng/ml (77.8%), and a Gleason score of 8-10 (59.6%) (Table 1).

**Table 1:** Demographics of the participants

Variables	Frequency (percentage)	Mean ± standard deviation
Age (years)		65.13 ± 6.84
≤65	86 (42.4%)	
>65	117 (57.6%)	
PSA level (ng/ml)		213.39 ± 543.20
<4	25 (12.3%)	
4-10	20 (9.9%)	
>10	158 (77.8%)	
Gleason score		8.01 ± 1.65
6 (Low grade)	16 (7.9%)	
7 (intermediate grade)	66 (32.5%)	
8-10 (High grade)	121 (59.6%)	

Of 203 patients, 126 (62.1%) and 77 (37.9%) had MRI and CT for the radiological diagnosis, respectively. The most common radiological finding was T3b in 104 (51.2%) cases, i.e., 51 (25.1%) cases were diagnosed on CT, and 53 (26.1%) cases were diagnosed on MRI. T4 was the second most common presentation on the radiological investigations in 43 (21.2%) cases (Table 2).

**Table 2:** Radiological presentation of prostate cancer

Stage	CT (n=77)	MRI (n=126)	Total (N=203)
Tx: primary tumor cannot be assessed	-	1 (0.5%)	1 (0.5%)
T1c: tumor identified by needle biopsy found in one or both sides, but not palpable	1 (0.5%)	-	1 (0.5%)
T2a: tumor involves one-half of one side or less	-	4 (2.0%)	4 (2.0%)
T2b: tumor involves more than one-half of one side but not both sides	2 (1.0%)	14 (6.9%)	16 (7.9%)
T2c: tumor involves both sides	3 (1.5%)	11 (5.4%)	14 (6.9%)
T3a: extraprostatic extension (unilateral or bilateral)	1 (0.5%)	19 (9.4%)	20 (9.9%)
T3b: tumor invades seminal vesicle(s)	51 (25.1%)	53 (26.1%)	104 (51.2%)
T4: tumor is fixed or invades adjacent structures other than seminal vesicles such as external sphincter, rectum, bladder, levator muscles, and/or pelvic wall	19 (9.4%)	24 (11.8%)	43 (21.2%)

The lymph node involvement was present in 87 (42.9%) and metastasis in 120 (59.1%) patients. Bones were the most common site for primary metastasis observed in 97 (47.8%) patients, followed by lungs in eight (3.9%) patients and non-regional lymph nodes in six (3.0%) patients. Non-regional lymph nodes in 22 (10.8%) patients and distant lymph nodes in 14 (6.9%) patients were the most common sites for secondary metastasis. Major tertiary metastatic sites were the lungs in four (2.0%) patients, the liver in four (2.0%) patients, and distant lymph nodes in four (2.0%) patients (Table 3).

**Table 3:** Metastasis of prostate cancer

Variable	Frequency (percentage)
Lymph node involvement	
N0: no positive regional nodes	16 (57.1%)
N1: metastases in regional node(s)	87 (42.9%)
Metastasis	
Present	120 (59.1%)
Absent	83 (40.9%)
Primary metastasis	
Bones	97 (47.8%)
Lungs	8 (3.9%)
Distant lymph nodes	5 (2.5%)
Non-regional lymph nodes	6 (3.0%)
Retroperitoneum	1 (0.5%)
Absent	86 (42.4%)
Secondary metastasis	
Bones	7 (3.4%)
Lungs	7 (3.4%)
Distant lymph nodes	14 (6.9%)
Non-regional lymph nodes	22 (10.8%)
Liver	5 (2.5%)
Absent	148 (72.9%)
Tertiary Metastasis	
Bones	2 (1.0%)
Lungs	4 (2.0%)
Liver	4 (2.0%)
Distant lymph nodes	4 (2.0%)
Non-regional lymph nodes	2 (1.0%)
Retroperitoneum	1 (0.5%)
Absent	186 (91.6%)

PSA >10 ng/mL was significantly associated with higher odds of metastasis compared to PSA <4 ng/mL on both unadjusted (OR=5.55, 95% CI: 2.18-14.15, p=0.0003) and adjusted analysis (AOR=4.98, 95% CI: 1.80-13.74, p=0.002). High Gleason score (8-10) was independently associated with metastasis (AOR=6.14, 95% CI: 1.50-25.14, p=0.01) (Table 4).

**Table 4:** Univariate and multivariate regression analysis of demographic factors with metastasis

Variable	Metastasis		Odds ratio	p-value	Adjusted odds ratio	p-value
	Present	Absent				
Age (years)						
<65	53 (61.6%)	33 (38.4%)	Reference	-	-	-
>65	67 (57.3%)	50 (42.7%)	0.83 (0.47-1.47)	0.532	0.96 (0.50-1.84)	0.905
PSA level (ng/ml)						
<4	7 (28.0%)	18 (72.0%)	Reference	-	-	-
4-10	15 (75%)	5 (25.0%)	0.86 (0.23-3.26)	0.821	0.81 (0.19-3.35)	0.768
>10	108 (68.4%)	50 (31.6%)	5.55 (2.18-14.15)	0.0003	4.98 (1.80-13.74)	0.002
Gleason score						
6 (Low grade)	3 (18.8%)	13 (81.3%)	Reference	-	-	-
7 (intermediate grade)	30 (45.5%)	36 (54.5%)	3.61 (0.94-13.87)	0.061	1.78 (0.42-7.61)	0.435
8-10 (High grade)	87 (71.9%)	34 (28.1%)	11.09 (2.97-41.36)	0.0003	6.14 (1.50-25.14)	0.01

The intermediate Gleason score had a lower incidence of lymph node involvement than the high Gleason score (OR=0.431 (0.231-0.804), (p=0.008), aOR=0.442 (0.233-0.839), (p=0.012)) (Table 5).

**Table 5:** Univariate and multivariate regression analysis of demographic factors with lymph node involvement

Variable	Lymph node involvement		Odds ratio	p-value	Adjusted odds ratio	p-value
	N_0_	N_1_				
Age (years)						
<65	42 (48.8%)	44 (51.2%)	1.803 (1.024-3.174)	0.041	1.689 (0.917-3.109)	0.092
>65	74 (63.2%)	43 (36.8%)	Reference	-	-	-
PSA level (ng/ml)						
<4	19 (76.0%)	6 (24.0%)	0.332 (0.126-0.876)	0.026	0.434 (0.149-1.260)	0.125
4-10	16 (80.0%)	4 (20.0%)	0.263 (0.084-0822)	0.022	0.348 (0.105-1.160)	0.086
>10	81 (51.3%)	77 (48.7%)	Reference	-	-	-
Gleason score						
6 (Low grade) *	0 (0%)	16 (100%)	-	-	-	-
7 (Intermediate grade)	44 (66.7%)	22 (33.3%)	0.431 (0.231-0.804)	0.008	0.442 (0.233-0.839)	0.012
8-10 (High grade)	56 (46.3%)	65 (53.7%)	Reference	-	-	-

## Discussion

This study provides a comprehensive analysis of the radiological staging and pattern of metastasis of prostate cancer in SKMC. The findings indicated a high burden of advanced disease at the time of diagnosis, with the majority of patients presenting with T3b (51.2%) and T4 (21.2%) stage prostatic tumors along with distant metastasis and lymph node involvement. Masood et al. reported that 29.2% had locally invasive and 21.3% had metastatic prostate cancer at presentation at a tertiary care hospital in Islamabad [13]. Jayasankar et al. reported that 42.9% of prostate cancers were diagnosed at the advanced stage, followed by 29.9% at the localized and 27.9% at the locoregional stage [14]. Sayan et al. reported prostate cancer data from the Middle East with 54% and 20% of patients having stage 4 and 3 disease at presentation, respectively [15]. PSA screening for prostate cancer has resulted in the detection of early-stage diagnoses and a reduction in the mortality rate. However, prostate cancer staging at diagnosis varies widely across the globe. Considerable disparities have been observed regarding the provision of screening, staging, and survival depending upon the age, ethnicity, education, income, and geographical region [16]. High-income countries provide widespread PSA screening, resulting in early-stage diagnosis, usually at the localized tumor stage. Conversely, lower middle-income countries (LMICs) report late diagnosis with the advanced staging of the disease, attributing to the lack of efficient screening programs and awareness of the disease.

Metastasis was present in most patients at the time of presentation, and bones were the most common site of metastasis, followed by lungs. Non-regional and distant lymph nodes were the most common sites for secondary metastasis. Gandaglia et al. reported a similar finding that the most common site for metastasis in prostate cancer was bone (84%), and the other sites for metastasis were distant lymph nodes (10.6%), liver (10.2%), and thoracic cavity (9.1%). The most common secondary metastasis sites in prostate cancer patients without bone involvement were distant lymph nodes (41.9%) [17]. Similarly, Shou et al. found that the most common site for the single metastasis in patients with stage IV prostate cancer was bone, with a median survival period of 30.494 months (p<0.001) [18].

A significant correlation of high PSA levels and high Gleason score with prostate cancer metastasis has been found. Patients with PSA levels >10 ng/ml and high-grade Gleason score (8-10) had a higher chance of prostate cancer metastasis. PSA level had a significant relation with the diagnosis, aggressive nature, and bone metastasis of prostate cancer. The higher cut-off value of PSA had the highest sensitivity, positive predictive value, and positive likelihood ratio for the metastasis of prostate cancer. Thomsen et al. reported that advanced T stage, higher PSA level, and higher level of Gleason grading group (GGG) were strongly associated with the metastasis of prostate cancer. Male patients with T1-T2 stage, PSA level 100-300 ng/ml, and GGG 1 had only 10% prostate cancer metastasis. T3-T4 stage, PSA level >500 ng/ml, and GGG 3 had 91% prostate cancer metastasis [19]. Similarly, Markowski et al. reported that the Gleason score was an independent risk factor for the distant metastasis of prostate cancer. A higher Gleason score was significantly associated with the development of prostate cancer metastasis (Gleason 7 vs. 6: hazard ratio (HR), 1.76; CI 1.14-2.73; p=0.011; Gleason 8-10 vs. 6: HR, 3.44; 95% CI 2.18-5.43; p≤0.001) [20]. These associations emphasized the value of PSA and Gleason scoring as critical resources for risk stratification and clinical choices.

A high-grade Gleason score had higher odds of lymph node metastasis of prostate cancer. Cheng et.al reported that lymph node positive patients had a higher preoperative PSA concentration (median: 8 ng/mL for lymph node negative patients, 21 ng/mL for lymph node positive patients; p<0.001) and a higher Gleason grade (5% of lymph node negative patients and 21% of lymph node positive patients had a Gleason grade ≥8; p=0.001) [21]. Xu et al. found that univariate analysis indicated GGG as a risk factor for pathological lymph node metastasis (p<0.001). Similarly, multivariate regression analysis suggested that in prostate cancer patients with GGG ≥3, the postoperative GGG was also a risk factor for the lymph node metastasis (OR 1.767; CI 1.061-2.943; p=0.029) [22]. Correlating the Gleason score with lymph node metastasis could assist in planning for the complete removal of the disease to proceed with either sentinel, extended, or pelvic lymphadenectomy and to prevent the recurrence of prostate cancer.

Prostate-specific membrane antigen (PSMA) PET/CT has emerged as a highly sensitive and specific imaging modality for staging and restaging prostate cancer. Unlike conventional CT or bone scintigraphy, PSMA PET/CT detects disease at lower PSA levels and identifies small-volume nodal and distant metastases that may be occult on routine imaging. It has demonstrated superior accuracy in evaluating biochemical recurrence, guiding targeted therapy, and refining radiation or surgical planning [23]. The integration of PSMA PET/CT into clinical practice has therefore improved risk stratification, detection of early metastatic spread, and individualized treatment decision-making, particularly in patients with high PSA levels or high-grade Gleason scores.

Limitations

The retrospective study design could lead to selection and information biases, as it depends on pre-existing medical records having probability of incomplete data. The single-centre study design could limit the generalizability of the data to a diverse population. The staging of prostate cancer was based only on radiological imaging without any correlation to pathological or surgical findings, potentially impacting the accuracy of the staging interpretation of the disease.

## Conclusions

This study provided an essential insight into the radiological representation and staging properties of prostate cancer, presenting a high proportion of patients with advanced disease, particularly with T3 b and T4 tumors. A significant proportion of patients had lymph node involvement and distant metastases at the time of diagnosis. The significant association between elevated PSA levels and higher Gleason scores with the metastasis of prostate cancer indicated the clinical relevance of PSA evaluation and the Gleason classification as predictive tools for disease progression and management. Educating health professionals and the general population about the significance of PSA screening, symptoms, and risk factors could lead to early diagnosis and treatment, thereby preventing disease progression. Furthermore, improving access to high-quality radiological imaging and multidisciplinary cancer care can significantly improve diagnostic precision and overall management of patients.
